# Preference reversal in intertemporal decision making

**DOI:** 10.3389/fpsyg.2024.1423615

**Published:** 2024-12-04

**Authors:** Yan-Bang Zhou, Kun Zhang, Hong-Kun Zhai, Qing Bao, Shanshan Xiao, Junhua Dang

**Affiliations:** ^1^Institute of Teacher Education, Ningxia University, Yinchuan, China; ^2^Faculty of Education, Tianjin Normal University, Tianjin, China; ^3^School of Journalism and Communication, Ningxia University, Yinchuan, China; ^4^Department of Psychology, Stockholm University, Stockholm, Sweden; ^5^School of Humanities and Social Sciences, Xi’an Jiaotong University, Xi’an, China

**Keywords:** preference reversal, intertemporal choice, gain, loss, response modes

## Abstract

This study examines asymmetric preference reversals in intertemporal decision-making by comparing gain and loss contexts across choice and bidding tasks. In the gain context, participants preferred smaller, sooner (SS) rewards in choice tasks but assigned higher valuations to larger, later (LL) rewards in bidding tasks. Conversely, in the loss context, they showed a preference for LL options in choice tasks but provided lower bids for SS options. Bidding tasks consistently required longer decision times than choice tasks, indicating greater cognitive demands during valuation processes. A real-world questionnaire involving 370 participants validated these findings across economic and health-related scenarios. These results underscore the role of task formats in shaping preferences, offering practical insights for refining strategies in behavioral decision-making and applied contexts.

## 1 Introduction

Decisions that involve trade-offs between outcomes occurring at different points in time are called intertemporal choice (Frederick et al., 2002; Scholten et al., 2024). Throughout our lives, we constantly make decisions about immediate actions versus those we postpone. Intertemporal choices are often involved in both positive and negative situations, such as spending on luxury goods now versus saving money for retirement and paying credit card debt regularly versus accumulating it. People in these future consumption decisions usually consider future consequences, hoping to gain immediate benefits and postpone losses. That is to say, they generally prefer smaller but sooner gains (vs. larger but later gains) as well as larger but later losses (vs. smaller but sooner losses) in a choice task (Berndsen and van der Pligt, 2001; Frederick et al., 2002; Thaler, 1981). Interestingly, individuals tend to respond quickly in binary choices, seemingly making intuitive judgments (Dhar and Gorlin, 2013; De Neys, 2023; Kahneman, 2011; Zhou et al., 2024). The outcome often reflects a comparative evaluation of options, where individuals determine a preference by weighing alternatives, akin to balanced judgment (Jiang et al., 2022).

However, individuals’ preferences can be elicited through various methods (Lempert and Phelps, 2016; Loomes and Pogrebna, 2017). In addition to the choice task, there are many other types of preference elicitation methods such as bidding, matching, fixed-sequence choice titration, and a dynamic “staircase” choice method (Attema and Brouwer, 2013; Hardisty et al., 2013; Rubaltelli et al., 2012). Among various methods, the bidding task stands out as particularly unique. Individuals provide a specific amount to represent their preference (Grether and Plott, 1979; Lichtenstein and Slovic, 1971). For instance, when booking a room online, guests who genuinely intend to stay must pay a deposit, involving different mental accounts (Thaler, 1985; Thaler, 1999). The bidding task yields a numeric value, typically requiring more time and making decisions more challenging, as if one is measuring with a precise scale (Dhar and Gorlin, 2013; Kahneman, 2011; Schkade and Johnson, 1989).

In the realm of risk decision-making, researchers have found that when college students faced p-bets (higher probability of winning a small amount) and $-bets (lower probability of winning a larger amount), they preferred p-bets in choice tasks while bidding higher on $-bets, revealing a preference reversal (Ball et al., 2012; Kim et al., 2012; Lichtenstein and Slovic, 1971; Schkade and Johnson, 1989). This preference reversal is not limited to the gain context; it has also been observed in the loss context. For instance, individuals preferred $-bets (lower probability of losing a large amount) in choice tasks, suggesting they perceived these options as less risky. Conversely, they bid lower on p-bets (higher probability of losing a small amount) in bidding tasks, indicating a perception of lower risk associated with these options (Lichtenstein and Slovic, 1973).

Preference reversal arises from the differing cognitive processes involved in these elicitation methods. Choice invokes more qualitative reasoning, such as the use of an ordering or sequence strategy which is cognitively easier than making explicit tradeoffs (Slovic, 1995). Tversky et al. (1988) proposed that ordering considerations loom larger in the procedure of choice than in the procedure of valuation. When individuals choose between two options, their mental procedure is analogous to weighing two objects by a balance. This comparison will produce a prominent effect, which means that the advantage of one dimension will be more prominent, thus making the decision follows the difference on the prominent dimension. For example, when an individual face two risk options (e.g., high probability to win a small amount vs. low probability to win a large amount or high probability to lose a small amount vs. low probability to lose a large amount), the probability dimension will be prominent due to loss aversion (Attema et al., 2013; Kim et al., 2012). Therefore, individuals tend to choose a p-bet (high probability to win a small amount) in the gain context because it can bring some gains more likely. They also tend to choose a $-bet (low probability to lose a large amount) in the loss context because it can avoid a loss more likely. However, when individuals bid on an option in a bidding task, their mental procedure is analogous to measuring an independent object with a caliper. When a dimension of the object (e.g., the amount of money of a risky option) is the same as the output dimension (i.e., how much money one would like to bid), this dimension of the object will be weighed more during the measuring. On the contrary, if a dimension of the object (e.g., the probability of a risky option) is different from the output dimension, individuals will need to translate this dimension into the output dimension, which makes individuals bear the cognitive burden and become more patience (Jiang and Dai, 2024). This effect is called the compatibility effect, which has been proved by many studies (Mellers et al., 1992; Tversky et al., 1990; Zhou et al., 2021, 2022). Therefore, individuals tend to bid higher on a $-bet in the gain context because it can probably bring more gains. They also tend to bid lower on a p-bet in the loss context because it indicates less losses.

According to Decision Field Theory (DFT), decision-making is characterized by the gradual accumulation of evidence (Busemeyer and Townsend, 1993). In choice tasks, individuals typically concentrate on a single salient dimension of the options presented, which facilitates quicker evidence accumulation and often results in more impulsive decisions (Frederick et al., 2002). Conversely, bidding tasks necessitate the consideration of multiple dimensions, leading to a more deliberate process of evidence accumulation and fostering greater patience in decision-making (Loewenstein and Prelec, 1993). This distinction is further supported by the Drift Diffusion Model (DDM), which illustrates that the complexity of a task influences the evidence accumulation process and shapes preferences (Ratcliff and McKoon, 2008). Together, DFT and DDM provide a comprehensive framework for understanding why individuals exhibit impulsivity in choice tasks while demonstrating greater patience in bidding tasks, thereby offering insights into the cognitive processes underlying these distinct decision-making contexts.

In intertemporal decision making, given loss aversion and the prevalence of impatience (Loewenstein and Prelec, 1993; Weber et al., 2007), most people prefer smaller but sooner gains (vs. larger but later gains) as well as larger but later losses (vs. smaller but sooner losses) in a choice task (Harris, 2012). However, their preference in the bidding task should be reversed due to the compatibility effect (Slovic et al., 1990; Tversky et al., 1988), because the output dimension and the money attribute of an intertemporal option are compatible when bidding. They focus on what they can obtain or lose without paying too much attention to the time delay. Therefore, people should bid higher on larger but later gains because it can bring more gains. They also should bid lower on smaller but sooner losses because it involves less losses. In sum, in the gain context, individuals’ preference should change from the smaller but sooner option (the SS option hereafter) in the choice task to the larger but later option (the LL option hereafter) in the bidding task. While in the loss context, individuals’ preference should change from the LL option in the choice task to the SS option in the bidding task. The occurrence of this preference reversal phenomenon would illustrate an asymmetry rule in intertemporal decisions.

Several studies have demonstrated various forms of preference reversal within the gain context of intertemporal decision-making (Xu et al., 2022a; Xu et al., 2022b). Tversky et al. (1990) first showed this phenomenon by presenting options of varied sums of money to be received at different times in the future. For instance, one option might offer $1,660 in 18 months (the SS option) and the other may offer $3,550 in 10 years (the LL option). Respondents were more likely to choose the SS option but place a higher value on the LL option (Zhou et al., 2021, 2022). However, to our best knowledge there is no study that has tested this kind of preference reversal in both gain and loss contexts. This study was designed to create an experimental scenario with symmetric gains and losses to test whether the preference reversal observed in the gain context could be replicated and whether an asymmetric preference reversal could be identified in the loss context. We expected that the proportion of the SS preference would be higher in the choice task than in the bidding task in the gain context. In the loss context, the preference would be reversed, such that the proportion of the SS preference would be lower in the choice task than in the bidding task. The proportion of the LL preference would be in an opposite pattern. Building on this foundation, the remainder of the paper outlines the methods in Section 2, presents the results in Section 3, discusses the implications of the findings in Section 4, and concludes with key insights and future directions in Section 5.

## 2 Methods

### 2.1 Participants

The statistical software G*Power was utilized to determine the required number of participants for a 2 (context: gain vs. loss) × 2 (preference elicitation method: choice vs. bidding) within-subject design, with *α* set at 0.05, power at 0.95, and an effect size f set at 0.25, which is a medium level of effect size commonly observed in preference reversal studies. The analysis indicated a required sample size of *N* = 44 participants. However, to enhance the robustness and generalizability of the findings, we recruited a total of 98 college students (78 females). This decision to exceed the minimum sample size aimed to account for potential variability in responses and allow for more comprehensive analyses of subgroups. The average age of participants was 19.66 years (SD = 1.24), and they received an average compensation of $11 for their participation. All participants provided informed consent, and the study was approved by the university’s ethics committee, adhering strictly to established regulations.

### 2.2 Apparatus

The display was a 17-inch ThinkPad LCD with a refresh rate of 60 Hz and a screen resolution of 1,024 × 768 pixels. Participants used the keyboard to respond.

### 2.3 Stimuli

Four intertemporal options were derived from Tversky et al.’s (1990) study. We subsequently added 26 intertemporal options based on these four options. Therefore, a total of 30 intertemporal options were used to form 15 pairs of intertemporal options. Each option represented a certain amount of monetary gain or loss after a certain period time. The stimuli were presented through the E-prime 3.0 software on the computer. For details of the stimuli, please refer to the following link: https://osf.io/s9cmt/?view_only=8bf1ef2948c647d9bb35daac2cdb1b5e.

### 2.4 Task

#### 2.4.1 Experimental tasks

The choice task and the bidding task were conducted within both the gain and loss contexts, resulting in a 2 (context: gain vs. loss) × 2 (preference elicitation method: choice vs. bidding) within-subject design. In the choice task in both contexts, each time participants chose between a SS option and a LL option. In the bidding task, each time participants bid for a single intertemporal option presented in the choice task. The order of the tasks and the presenting location of the option were all counterbalanced.

In the choice task, each pair of intertemporal options was presented twice, with the left–right placement of options switching between presentations. Therefore, there were two blocks consisting of 60 trials in total (30 in the gain context and 30 in the loss context) in the choice task. In the bidding task, each option was presented once and there were also two blocks consisting of 60 trials in total (30 in the gain context and 30 in the loss context). Participants used a number pad on the keyboard to enter their price without time limitation. There were two practice trials for both tasks prior to the main experiment to ensure participants’ understanding of the instructions.

#### 2.4.2 Reward plan

The gain trials and the loss trials were incentivized in a compatible way. Participants were informed at the start of the experiment that they possess 10,000 units of virtual currency for making decisions within the game. At the game’s end, we would randomly select one decision from each of the four tasks and calculate the rewards based on a certain proportionate discount. We would not disclose the full details of how rewards are calculated until the experiment ended. This approach was taken to avoid influencing the participants’ decisions. In the case of the choice task, one trial was randomly selected. According to the participant’s choice in the selected trial, the outcome was discounted as the actual reward at the end of the experiment. In the case of the bidding tasks, the reward calculation referred to the Becker–DeGroot–Marschak (BDM) method (Becker et al., 1964), yet it was not identical to the BDM method. After a bidding trial was randomly selected, a random number was generated between 0 and the outcome of the selected option. In the gain bidding task, if a participant’s bid exceeded a random number, they acquired the option, thereby securing future gains and paying the bid amount. If the bid fell below the random number, no payment was required, and participants retained their bid. For instance, in the option “get 1,600 yuan in 1.5 years,” if the random number was 500 and the bid was 800, participants had to immediately pay 800 units of virtual currency to secure the option to “get 1,600 yuan in 1.5 years.” If the bid is below the random number, no payment was required. In contrast, in the loss bidding task, a bid above the random number avoided future loss. For instance, with the option “lose 1,600 yuan in 1.5 years,” if the random number was 1,000 and the bid was 800, participants did not pay immediately but had to accept the future loss of “1,600 yuan in 1.5 years.” If the random number was below the bid, immediate loss of the bid amount was required. For instance, in the “lose 1,600 yuan in 1.5 years” option, if the random number was 500 and the bid was 800, participants had to immediately pay 800 units of virtual currency. Reward and waiting times for participants were determined by their current holdings of virtual currency and the options they possessed. Finally, the virtual currency owned by participants was multiplied by a coefficient of 0.01, and this adjusted amount was immediately disbursed as the actual reward. Similarly, the delay and outcome in the options owned by participants were multiplied by a coefficient of 0.01. For example, a 1.5-year delay was equivalent to roughly 5 days; 10,000 units of virtual currency correspond to ¥100. The average reward at the end of the experiment was ¥90.6 ($11).

### 2.5 Preference measurement

In the intertemporal choice task, participants chose between two options that differed in the timing of rewards or losses. One option provides a smaller outcome with a shorter delay (SS), while the other offers a larger outcome with a longer delay (LL). For each pair of intertemporal options, we categorize participants’ responses into three groups: choosing SS both times, choosing each option once, or choosing LL both times. Choosing SS both times indicates a preference for shorter delays, while choosing LL both times indicates a preference for longer delays. The preference is measured by the ratio of SS to LL choices, where a higher ratio of SS choices signifies a stronger inclination toward immediate outcomes, and a higher ratio of LL choices indicates a preference for delayed outcomes. This categorization applies to both gain and loss contexts.

In the intertemporal bidding task, participants place a bid on a single outcome with a specific delay, reflecting their valuation of rewards or costs occurring at different times. In the gain context, we classify the bidding responses based on whether participants bid higher on SS, bid equal amounts for both options, or bid higher on LL. Bidding higher on SS indicates a preference for shorter delays, while bidding higher on LL indicates a preference for longer delays. In the loss context, responses are categorized as bidding lower on SS, bidding equal amounts, or bidding lower on LL. Bidding lower on SS suggests a preference for immediate costs, while bidding lower on LL indicates a preference for delaying larger losses. The preference in the bidding task is measured by analyzing bid amounts at different delay times for both gains and losses.

Through these two tasks, we analyze participants’ preferences for SS and LL by recording their choice and bidding ratios, which serve as our primary dependent variables.

### 2.6 Procedure

**Figure 1:**
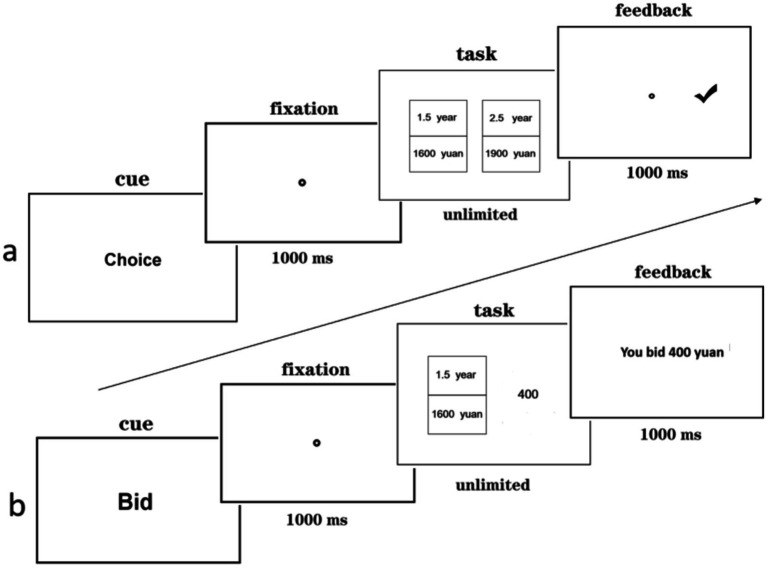
The procedure for presenting the choice **(A)** and the bidding task **(B)**.

Firstly, participants provided their informed consent. Then the instructions were presented on the computer screen, and following successful completion of the practice trials, the main experiment commenced. In the choice task, “Choice” was displayed on the center of the screen for 1,000 ms, followed by a calibration point. Participants fixated on the calibration point and pressed the space bar to skip to the next screen. Then, two intertemporal options appeared on the left and right sides of the screen. Participants browsed the options and made their decision by pressing the “F” key for the left option and “J” key for the right option. After making a choice, feedback was provided on the screen (see Figure 1A).

In the bidding task, “Bid” was displayed in the center of the screen for 1,000 ms, followed by a calibration point. Participants fixated on the calibration point and pressed the space bar to skip to the next screen. Then, an intertemporal option appeared on the left side of the screen, and participants made a subjective value assessment of the option. They used the number pad on the keyboard to enter the specific amount of money they would be willing to spend on the intertemporal option in the gain context and the specific amount of money they would be willing to spend to avoid the loss indicated in the intertemporal option. The input numbers were displayed on the right side of the intertemporal option simultaneously. After pressing the “Enter” key to submit the bidding price, feedback was shown on the screen. If participants made a mistake, they could use the backspace key to modify it. Once the “Enter” key has been pressed, this trial ended (as shown in Figure 1B). Participants were asked to choose or bid based on the values presented in the stimuli.

At the end of the experiment, participants receive their rewards, which comprise earnings from two gain trials and two loss trials, along with any remaining virtual currency. We then calculate the actual rewards, including delays, with a 0.01 discount, and transfer the earnings or deduct any losses through WeChat.

## 3 Results

### 3.1 Preference reversal

We defined the independent variables as task type (choice and bidding) and context type (gain and loss), and the dependent variable as the proportion of preference for SS. Subsequently, we conducted a two-factor repeated measures analysis of variance. The results indicated a significant main effect of context type, *F*(1, 97) = 4.81, *p* = 0.031, $\eta_{p}^{2}$ = 0.05, as well as a significant interaction effect between task type and context type, *F*(1, 97) = 166.29, *p* < 0.001, $\eta_{p}^{2}$ = 0.63. The main effect of task type was not significant, *F*(1, 97) = 1.27, *p* = 0.263, $\eta_{p}^{2}$ = 0.01.

**Figure 2:**
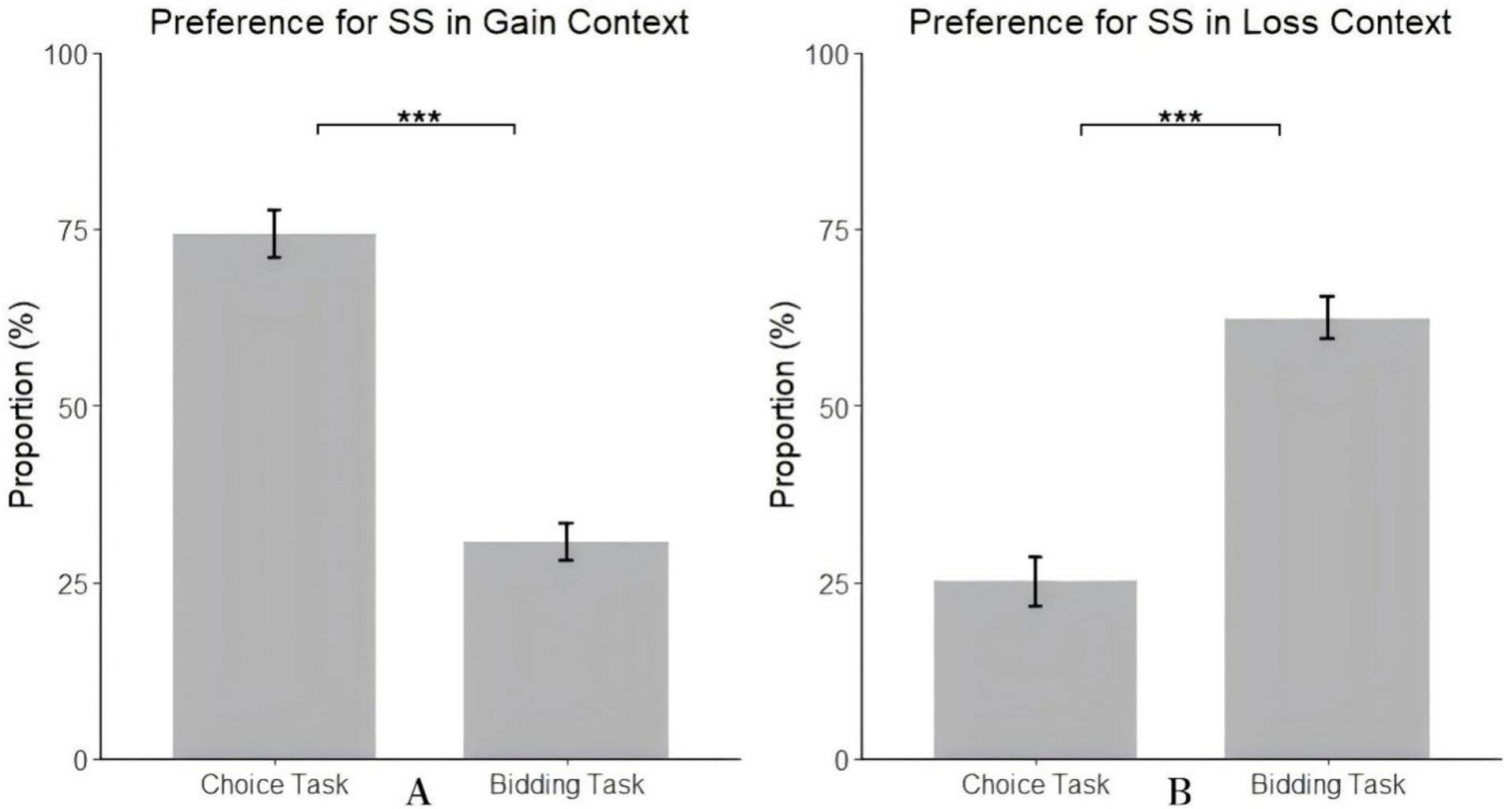
The proportion of SS in the gain context **(A)** and in the loss context **(B)**.

Simple effect analysis revealed significant differences in individuals’ preference for the SS between choice and bidding tasks in the gain context, *F*(1, 97) = 119.93, *p* < 0.001, $\eta_{p}^{2}$ = 0.55. Specifically, in the choice task, individuals’ proportion of choosing the SS (74.29%) was significantly higher than the proportion of bidding higher on the SS in the bidding task (30.74%) (see Figure 2A). Similarly, in the loss context, significant differences in individuals’ preference for SS between choice and bidding tasks were observed, *F*(1, 97) = 72.05, *p* < 0.001, $\eta_{p}^{2}$ = 0.42. In the choice task, individuals’ proportion of choosing SS (25.10%) was significantly lower than the proportion of bidding lower on the SS in the bidding task (62.38%) (see Figure 2B).

We defined the independent variables as task type (choice and bidding) and context type (gain and loss), and the dependent variable as the proportion of preference for the LL. Subsequently, we conducted a two-factor repeated measures analysis of variance. The results revealed a significant main effect of task type, *F*(1, 97) = 11.47, *p* = 0.001, $\eta_{p}^{2}$ = 0.11, and a significant interaction effect between task type and context type, *F*(1, 97) = 120.48, *p* < 0.001, $\eta_{p}^{2}$ = 0.55. The main effect of context type was not significant, *F*(1, 97) = 1.62, *p* = 0.206, $\eta_{p}^{2}$ = 0.02.

**Figure 3:**
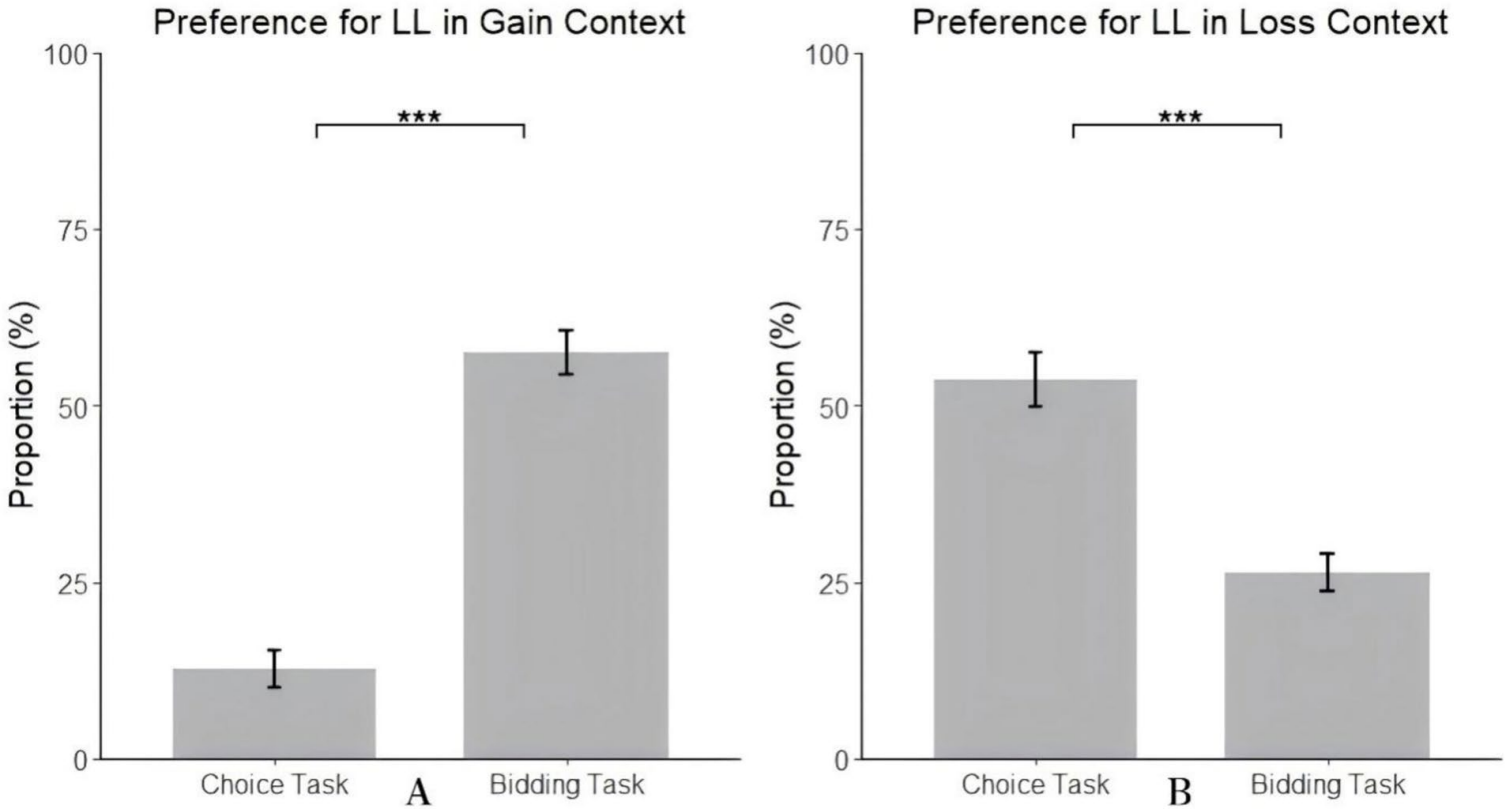
The proportion of LL in the gain context **(A)** and in the loss context **(B)**.

Simple effect analysis showed significant differences in individuals’ preference for the LL between choice and bidding tasks in the gain context, *F*(1, 97) = 134.69, *p* < 0.001, $\eta_{p}^{2}$ = 0.58. Specifically, in the choice task, individuals’ proportion of choosing LL (12.65%) was significantly lower than the proportion of bidding higher on the LL in the bidding task (57.55%) (see Figure 3A). Similarly, in the loss context, significant differences in individuals’ preference for the LL between choice and bidding tasks were observed, *F*(1, 97) = 36.88, *p* < 0.001, $\eta_{p}^{2}$ = 0.28. In the choice task, individuals’ proportion of choosing the LL (53.67%) was significantly higher than the proportion of bidding lower on LL in the bidding task (26.39%) (see Figure 3B).

### 3.2 Reaction time

To investigate the average reaction time of participants on different tasks in both the gain and the loss contexts. We defined the independent variables as task type (choice and bidding) and context type (gain and loss), and the dependent variable as reaction time. Subsequently, we conducted a two-factor repeated measures analysis of variance. The results revealed a significant main effect of task type, *F*(1, 97) = 198.76, *p* < 0.001, $\eta_{p}^{2}$ = 0.67. The main effect of context type [*F*(1, 97) = 0.97, *p* = 0.326, $\eta_{p}^{2}$ = 0.01] and the interaction effect between task type and context type [*F*(1, 97) = 2.72, *p* = 0.102, $\eta_{p}^{2}$ = 0.03] were not significant. Participants spent more time in the bidding task (*M* = 7,692 ms, *SD* = 3,306) than in the choice task (*M* = 3,329 ms, *SD* = 1,653). Because the RT data was not normally distributed (1-sample KS test, *p*s < 0.001) but positively skewed in all four tasks, the above analyses may have been biased. Therefore, we conducted a 5,000-run permutation test that does not require a normality assumption and is suitable for the non-normal RT data. The outcomes aligned with parametric findings, such that the main effect of task type was significant, *p*_permutation_ < 0.001. The main effect of context type (*p*_permutation_ = 0.339) and the interaction effect between task type and context type (*p*_permutation_ < 0.098) were not significant.

## 4 Discussion

In the context of intertemporal decision-making, individuals exhibited asymmetric preference reversals between gain and loss conditions. To be specific, in the gain context, individuals chose more SS options and bid higher on LL options, while in the loss context, individuals chose more LL options and bid lower on SS options. These shifts reflect a reversal in preferences: in the gain context, individuals switched from favoring SS options in choice tasks to LL options in bidding tasks, while in the loss context, they reversed from LL preferences in choice tasks to SS in bidding tasks. Furthermore, the bidding task required significantly more time to complete than the choice task in both gain and loss conditions.

Similar to preference reversal in risk decision making, theoretically, the p-bet or the SS option cannot be simultaneously better and worse than the $-bet or the LL option. Individuals’ preferences should not change with different measurement methods. For example, when measuring weight, a pan balance or spring scale should yield the same object ranking. The two measurement procedures should produce the same ordering (sequence) (Tversky and Thaler, 1990). However, preference reversal research has shown that different elicitation methods can alter the relative weight of attributes, producing different preference orderings. Preference reversal mainly breaks program invariance (Schkade and Johnson, 1989; Tversky et al., 1990; Hinvest et al., 2014), which is mainly caused by the prominent effect and the compatibility effect (Tversky and Thaler, 1990). It seems that human beings have a limitation of decision, suggesting that human beings’ decisions may be easy to be misled. This provides theoretical guidance for the irrationality of decision making.

In the choice task, the weight of attributes is similar to dictionary type (close to all or nothing) regardless of the context (gain or loss), which causes the most important attribute to be weighed more (Slovic et al., 1990; Tversky et al., 1988). In intertemporal decision-making, people tend to discount future emotions, preferring immediate pleasurable outcomes and deferring painful experiences. Accordingly, individuals prefer to choose small sooner gains and large later losses (Berndsen and van der Pligt, 2001; Loewenstein and Prelec, 1993; Weber et al., 2007). However, the weight of a stimulus can also increase due to the compatibility of response types (Slovic et al., 1990). It can be found in life experience, for example, the button sequence of factory boilers is consistent with the sequence of boilers. Due to this design, workers can control the equipment as fast as they can. For another example, in the second-hand car market, people pay attention to the price of the car firstly, while in the new car market, people pay more attention to other attributes. Similarly, regardless of the context, the output is bidding price, which is a dimension compatible with the amount of money in an intertemporal option. Therefore, the weight of the amount attribute increases.

We are interested in the time required to make these decisions for two reasons. First, it provides an approximate measure of the overall effort (Dai et al., 2018). Second, it allows us to examine specific ideas about the choice and bidding response modes. Because choice consists of two options, we might expect the time required to choose to be greater than the time required to make a single judgment. However, the results of the reaction time analysis showed the opposite. On average, one trial of the bidding task took 4 s longer than one trial of the choice task. This might be due to the difference in response input. However, it is unlikely that pressing one or two buttons could account for an increase of more than 4 s. The more likely reason is that it takes more time to evaluate the value of the options, rather than to type one or two more numbers. Actually, the finding that the bidding task takes longer is consistent with previous research (Schkade and Johnson, 1989). Researchers suggest that the cognitive resources required for the two tasks are different (Hinvest et al., 2014; Hunt et al., 2014; Loomes and Pogrebna, 2017). The decision making process of evaluating value is more complicated and demanding than choosing. Thus, time constraints may increase cognitive pressure on individuals during bidding tasks. Time pressure often forces individuals to speed up their evaluations, which can lead to greater preference inconsistency or reversal. Future research could better control the time factor to more accurately assess its impact on decision-making behavior.

The differences in decision time and assumed effort in the choice task and the bidding task may be related to the presentation features of these two tasks as well as individual differences in numeracy. The choice task presents a pair of options each time, while the bidding task presents a single option each time. Presenting two options simultaneously increases the tendency of order reasoning, which is cognitively easier than making explicit tradeoffs and thus takes less time (Slovic, 1995). Further, binary choice may also make people more impulsive to make decision (Figner et al., 2010). The effect of these features should be especially strong for those with lower numeracy because they are not confident with their number skills and thus tend to follow the intuition and make a quick decision. If the options in the choice are presented serially, with each option one time, and the choice decision is made after all options have been presented, this presentation may stimulate individuals to make explicit tradeoffs, thus canceling the preference reversal.

Preference reversals due to differences in response modes are common in everyday life. Previous research indicates that preference reversals often occur in health-related decisions when participants are presented with varying question frames (Bleichrodt and Pinto Prades, 2009), and similar phenomena have been observed in food-related decision-making (Chen et al., 2020). Additionally, in consumer contexts, choice deferral and emotional states, such as hope, have been found to influence preferences, especially when individuals perceive incomplete or ambiguous information (Wei et al., 2021). To validate these findings, we developed a comprehensive questionnaire comprising 4 real-life scenarios, including both choice and bidding questions, to assess preferences in economic and health domains. Examples of scenarios included questions such as, “If you are considering a discount, which option would you prefer?” and “If recharging a membership card, how much would you be willing to recharge?” The questionnaire was administered to 370 participants, and the results aligned closely with prior laboratory studies, suggesting broad applicability to fields such as marketing and negotiation.

These findings underscore the limitations of choice-based decision methods, as human biases and inconsistencies often emerge in choice tasks, highlighting the inadequacy of relying solely on choice-based methods for decision-making. Recognizing these limitations enables the development of strategies to reduce judgment errors, shape consumer preferences, and improve negotiation outcomes. Furthermore, our findings suggest that bidding tasks may capture subtle differences in value perception, providing a more accurate reflection of participants’ underlying preferences that might be overlooked in choice tasks. Thus, selecting suitable response modes is essential not only for enhancing the accuracy and effectiveness of decision-making insights but also for offering new perspectives in related research fields.

## 5 Conclusion

This study demonstrates that preference reversals in intertemporal decision-making are influenced by context (gain vs. loss) and task type (choice vs. bidding). Individuals tend to favor smaller sooner (SS) options in choice tasks within the gain context and larger later (LL) options in the loss context, but reverse these preferences in bidding tasks. Reaction time data indicates that bidding tasks require greater cognitive effort due to the explicit value assessment involved. These findings highlight the compatibility effect and the complexities of intertemporal preferences. This research enhances our understanding of how task formats affect attribute weighting and decision processes, emphasizing the need for appropriate response modes in preference elicitation. Future studies should examine the implications of these reversals in practical settings, particularly in financial and health-related decisions where decision consistency is crucial.

## Data Availability

The datasets presented in this study can be found in online repositories. The names of the repository/repositories and accession number(s) can be found at: https://osf.io/s9cmt/?view_only=8bf1ef2948c647d9bb35daac2cdb1b5e.
