# Supplementary material for: Preference reversal in intertemporal decision making
Source: Front Psychol. 2024 Dec 4;15:1423615. doi: 10.3389/fpsyg.2024.1423615 (PMC11652196; doi:10.3389/fpsyg.2024.1423615)
Supplement: Supplementary file 1 [file Table_1.DOCX]

**Questionnaire**

Hello! Thank you for participating in this survey. The following questions are designed to understand your preferences and decision-making tendencies. This survey is anonymous, and all data will be used exclusively for academic research. Your participation is voluntary, and you may withdraw at any time without penalty. We appreciate your support and cooperation!

Item A: Weight Loss Preferences

If you are considering weight loss, which option would you prefer?

☐ A. Using a weight-loss medication with no side effects, expected to result in a 5 kg loss in one month.

☐ B. Participating in a gym-based weight loss program, expected to achieve a 6 kg loss in one year.

If you are considering weight loss, how much would you be willing to pay per month for this medication with no side effects? (Estimated effect: 5 kg loss in one month)

Answer: ______ USD/month

If you are considering weight loss, how much would you be willing to pay per month for a gym membership? (Estimated effect: 6 kg loss in one year)

Answer: ______ USD/month

Item B: Savings Preferences

If you are considering making a deposit, which option would you prefer?

☐ A. A low-interest current account that allows for withdrawals at any time.

☐ B. A high-interest fixed-term deposit that can be accessed after one year.

If you are considering making a deposit, what estimated percentage of your assets would you consider allocating to the current account?

Answer: ______ %

If you are considering making a deposit, what estimated percentage of your assets would you consider allocating to the fixed-term deposit? (Accessible after one year)

Answer: ______ %

Item C: Certification for Promotion

If you need to obtain a certificate for promotion, which learning option would you prefer?

☐ A. A short-term training program that can be completed in one month, providing a certificate but limited skill enhancement.

☐ B. A long-term training program that takes six months to complete, providing a certificate and substantial skill improvement.

If you need to obtain a certificate for promotion, how much would you be willing to spend on a short-term training program? (Limited skill enhancement, can be completed in one month)

Answer: ______ USD

If you need to obtain a certificate for promotion, how much would you be willing to spend on a long-term training program? (Substantial skill improvement, takes six months to complete)

Answer: ______ USD

Item D: Membership Card Recharge for Discounts

If you are considering purchasing a product that requires recharging a membership card for discounts, which discount would you prefer?

☐ A. Recharge the membership card now to receive a 10% discount.

☐ B. Wait for a promotion in 1.5 months (pending availability) to receive a 20% discount.

If you are considering recharging a membership card to receive an immediate 10% discount, how much would you be willing to recharge?

Answer: ______ USD

If you are considering recharging a membership card to receive a 20% discount during a promotion in 1.5 months, how much would you be willing to recharge?

Answer: ______ USD

Thank you for your participation! All responses will remain confidential.
